# D-Glucosamine Conjugation Accelerates the Labeling Efficiency of Quantum Dots in Osteoblastic Cells

**DOI:** 10.1155/2014/821607

**Published:** 2014-03-24

**Authors:** Kazunari Igawa, Ming-Fang Xie, Hideki Ohba, Shizuka Yamada, Yoshihiko Hayashi

**Affiliations:** ^1^Department of Cariology, Nagasaki University Graduate School of Biomedical Sciences, Nagasaki 852-8588, Japan; ^2^Biotechnology Division of Care Four Company Ltd., Kokura-Kitaku, Kitakyushu 802-0071, Japan; ^3^Measurement Solution Research Center, National Institute of Advanced Industrial Science and Technology, Tosu, Saga 841-0052, Japan

## Abstract

Quantum dots (QDs) are useful imaging tools in the medical and biological fields due to their optical properties, such as a high fluorescence intensity, remarkable resistance to photobleaching, broad absorption spectra, and narrow emission spectra. This is the first study to investigate the uptake of carboxylated QDs conjugated with D-glucosamine (core size: approximately 3 nm, final modified size: 20–30 nm) into cultured osteoblastic cells. The QDs attached to the cell surface and were transported into the cytoplasm within approximately three hours of culture, whose process was clearly demonstrated using specific fluorescent staining of the cell membrane. Although the intranuclear distribution was not observed, a dramatic decrease in the transfer of quantum dots into the cytoplasm was recognized after approximately seven days of culture. Other interesting phenomena include the escape of the quantum dots from lysosomes in the cytoplasm, as confirmed by the merging of both QD fluorescence and specific fluorescent staining of lysosomes in the cytoplasm. These findings suggest that D-glucosamine conjugation enhances proton absorption in acid organelles and promotes the lysosomal escape of QDs.

## 1. Introduction

Cationic polymers with a large number of primary amine groups, such as polyamidoamine (PAMAM) dendrimer, polyethylenimine (PEI), and chitosan, are widely used in development of gene delivery carriers to promote cellular uptake via electrostatic interactions between positive and negative charges on the cell membrane [1–4]. Furthermore, these polymers are thought to have a strong pH-buffering capacity that enhances proton absorption in acid organelles and osmotic pressure buildup across the organelle membrane. These processes in turn promote the endosomal escape and release of genes into the cytoplasm [5, 6]. Quantum dots (QDs) are useful imaging tools for cellular labeling and monitoring due to their optical properties, such as a high fluorescence intensity, remarkable resistance to photobleaching, broad absorption spectra, and narrow emission spectra [7, 8]. Recently, QDs have been modified with PAMAM dendrimers for the efficient labeling of mesenchymal stem cells (MSCs). The uptake efficiency and cytosolic distribution of QDs in primary cultured MSCs are increased by modification of the PAMAM dendrimer [9]. However, it has not yet been investigated whether the chitosan monomer (D-glucosamine) accelerates the uptake of modified QDs into cells.

The aim of this paper was to label osteoblastic cells by QDs for* in vitro* imaging. D-glucosamine hydrochloride is an extremely low molecular bioactive material with a potential buffering capacity. We synthesized carboxylated QDs to conjugate with D-glucosamine in order to enhance both cellular uptake and endosomal escape in osteoblastic cells. The cellular uptake efficiency and intracellular distribution of D-glucosamine-conjugated QDs were investigated.

## 2. Materials and Methods

### 2.1. Synthesis of CdSe Core Nanocrystals (NCs)

Cadmium selenide (CdSe) NCs were synthesized and purified according to the method of Z. A. Peng and X. Peng [10]. Following purification, the vacuum-dried CdSe NCs were dissolved in chloroform and kept in the dark.

### 2.2. Carboxylation of QDs

Water-soluble CdSe core NCs were obtained using mercaptosuccinic acid as a surface-modifying agent. Briefly, 20–30 mg of NCs was dissolved in chloroform, after which 3 mL of dimercaptosuccinic acid (Sigma, dissolved in 150 mM PBS, pH 7.3) was added to 6 mL of NCs in chloroform. Water containing water-soluble CdSe NCs was carefully decanted and subjected to ultrafiltration to remove free (nonreacted) dimercaptosuccinic acid. The ultrafiltration was carried out using a concentrator (Vivaspin-6/20, Sartorius AG, Gottingen, Germany).

### 2.3. Conjugation of Water-Soluble CdSe Core NCs with D-Glucosamine

The water-soluble NCs were further conjugated with D-glucosamine. Carbodiimide chemistry was applied for conjugation, using N-(3-dimethylaminopropyl)-N′-ethylcarbodiimide hydrochloride (EDC) as a zero-length crosslinker. Briefly, 50 *μ*L of carboxylated QDs was mixed with 1 mL of D-glucosamine solution in PBS. Then, 100 *μ*L of 0.1 M EDC stock solution in water was added to the mixture. Subsequently, the mixture reacted while stirring mildly for 24 hours at 4°C.

### 2.4. Cell Culture and Cellular Uptake Studies

The osteoblastic cell line (NOS-1 cells [11]) from human osteosarcoma was purchased from Riken Cell Bank (Tsukuba city, Japan) and seeded in a 65 mm culture dish at a density of 1 × 10^5^ cells in *α*-MEM containing 10% fetal bovine serum and cultured in a humidified incubator at 37°C in an atmosphere of 5% CO_2_ and air. The subconfluent monolayer was passaged via trypsinization (trypsin-EDTA, Gibco Lab). The NOS-1 cells were seeded in a 35 mm glass-bottom culture dish (FluoroDish, World Precision Instruments, FL, USA) at a density of 6 × 10^5^ cells for three, six, and 24 hours of incubation or a density of 3 × 10^5^ cells for seven days of incubation. The final concentration of D-glucosamine conjugated with QDs was determined and adjusted to 0.05% in *α*-MEM after preliminary experiments regarding cell growth and QD uptake were performed. In the cells cultured for seven days, normal *α*-MEM was replaced three days after two days of incubation with conjugated QDs.

### 2.5. Fluorescent Staining of the Cell Membrane and Organelles

For cell membrane staining, fluorescent dye {CellMask (C10045), Life Technologies, CA, USA} was used. After removing the culture medium from the dish, the dish was covered with 0.5 mL of staining solution {1 *μ*L of original solution per 1 mL of PBS(+)} for five minutes in a humidified incubator at 37°C. After removing the staining solution, the culture dish was rinsed with PBS(+) three times and covered with PBS(+) for observation. For cell organelle staining, fluorescent dye {Organelle-ID RGB reagent I (ENZ-53007), ENZO Life Sciences International, PA, USA}, was used. After removing the culture medium from the dish, the dish was covered with 0.5 mL of staining solution {2 *μ*L of original solution per 1 mL of PBS(+)} for 30 minutes in a humidified incubator at 37°C. Subsequently, the culture dish was similarly washed and covered with PBS (+).

### 2.6. Confocal Laser Microscopy

Following cell membrane or organelle staining, the cells in 35 mm glass-bottom culture dishes were cultured in a 5% CO_2_ incubator (H301-TC1-HMTC, Okolab S.r.L., NA, Italy) for fluorescence observation then three-dimensionally analyzed (slicing width of cells: 1.5 *μ*m) using a confocal laser microscope (TCS SL, Leica Microsystems GmbH, Wetzlar, Germany) to detect QD-glucosamine conjugates delivered into the cells at different time intervals. The approximate fluorescence excitation/emission maxima for imaging of the QDs, cell membrane, and lysosomes were 385/525, 554/567, and 543/667, respectively.

## 3. Results

**Figure 1:**
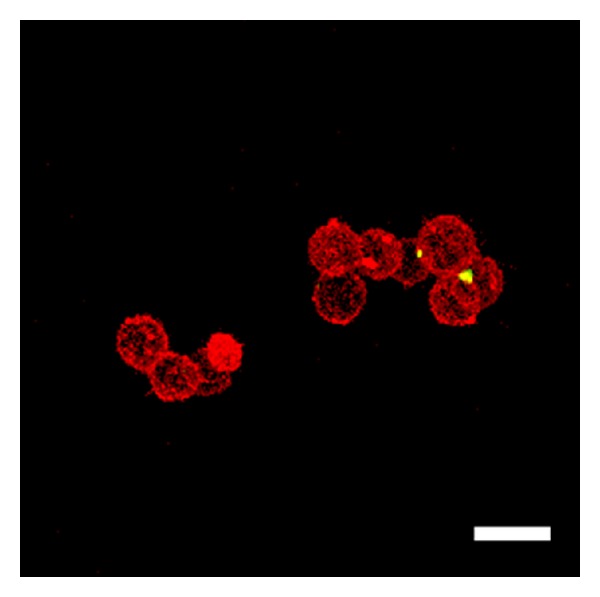
QD (high luminance of yellow-green fluorescence) inside the cell membrane (orange fluorescence) after three hours of culture. Scale bar = 15 *μ*m.

**Figure 2:**
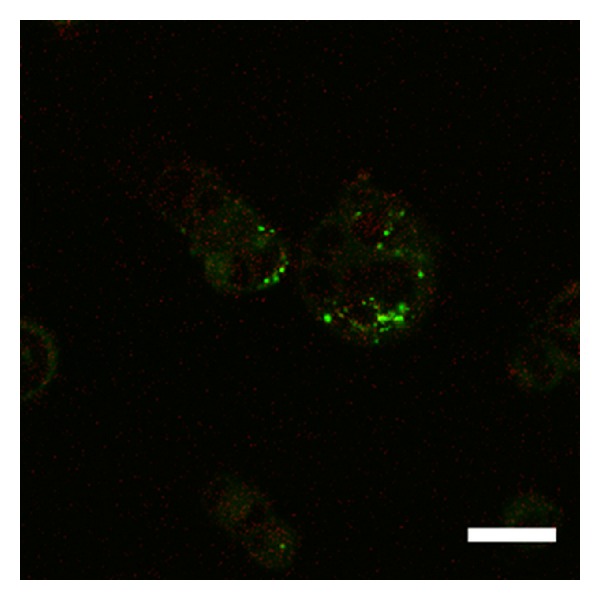
Many QDs (green fluorescence) are labeled inside the cells after one day of culture with D-glucosamine. Note the lack of overlap between the QDs and lysosomes (fluorescent red dots). Scale bar = 15 *μ*m.

**Figure 3:**
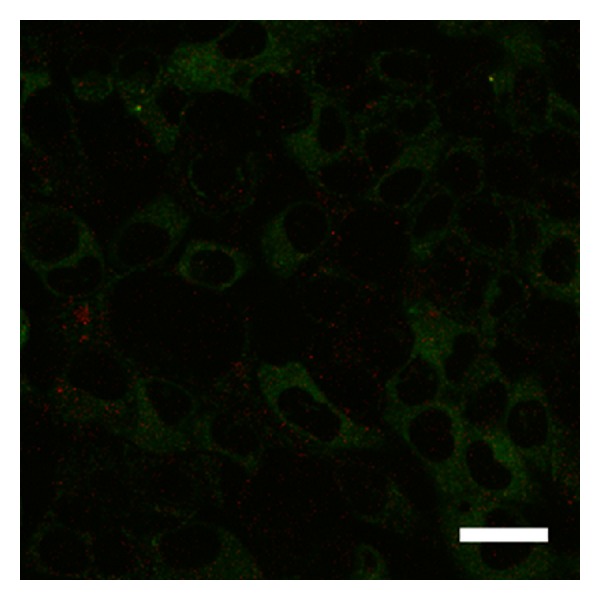
QD (green fluorescence) inside the cell after seven days of culture with D-glucosamine. Note the decrease in the number of QDs compared to that observed after one day of culture (Figure 2). Scale bar = 15 *μ*m.

**Figure 4:**
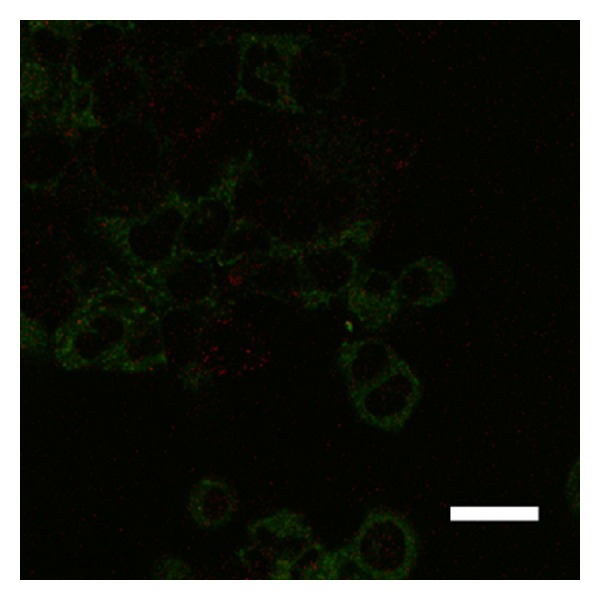
QD (green fluorescence) inside the cell after one day of culture without D-glucosamine. Note the partial overlap between the QDs and lysosomes (fluorescent red dots). Scale bar = 15 *μ*m.

**Figure 5:**
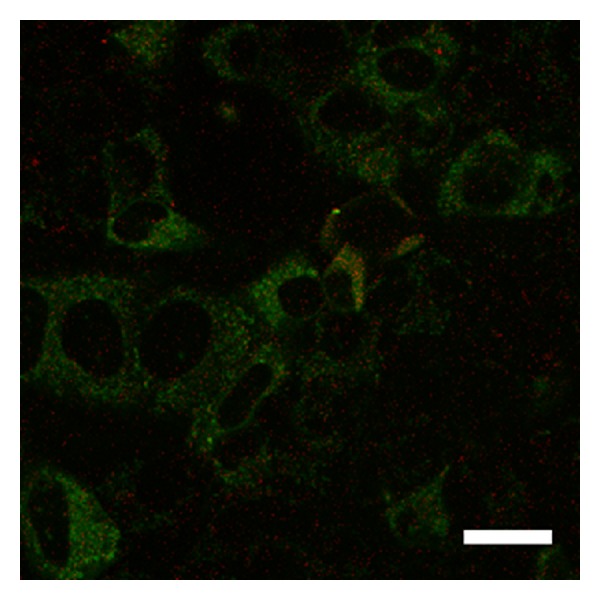
QD (green fluorescence) inside the cell after seven days of culture without D-glucosamine. Note the partial overlap between the QDs and lysosomes (fluorescent red dots). Scale bar = 15 *μ*m.

Over two hours, the stable observation of vital cells and fluorescence from QDs was possible using the 5% CO_2_ incubator. As the yellow-green fluorescent brightness from QDs was extremely strong, it was easy to differentiate these molecules from other cellular elements. The cell membrane staining and confocal microscopic imaging easily provided observations of the QD uptake into the cells. After three hours of culture, QDs were observed inside the cells (Figure 1). After one day of culture, many QDs were taken up into the cells (Figure 2). After seven days of culture, the distribution of QDs was dramatically decreased inside the cells (Figure 3). After seven days of culture, the intranuclear distribution of QDs was not observed. After one day (Figure 4) and seven days (Figure 5) of culture in the control group (cells treated without conjugating with D-glucosamine), only a few QDs were observed. After merging the fluorescent images of the QDs and lysosomes, no overlap was observed between these two structures in the experimental group (Figure 2). However, partial overlap was detected in the images for the QDs and lysosomes in the control group (Figures 4 and 5).

## 4. Discussion

D-glucosamine is used as an effective medicament in various fields of medicine and dentistry. For example, it is an attractive candidate for adjunctive therapy in patients with arthritis [12]. D-glucosamine also has a significant antipain effect in patients with osteoarthritis, a disease with low expectations of the value of treatment [13, 14]. The present study documents another biological action of D-glucosamine, that is, the dramatic increase of the cellular uptake of QDs via attachment with the cell membrane due to a positive charge and the biocompatibility of conjugated D-glucosamine. This phenomenon was confirmed in control experiments, which clearly indicated that nonconjugated QDs have difficulty entering cells. Furthermore, additional interesting findings include the escape of QDs from lysosomes inside cells, as confirmed with the observation of merged fluorescence of both QDs and lysosomes. This is the first study to observe the stability and protection of intracellularly distributed QDs following the application of the monomer type of chitosan, D-glucosamine. The proton sponge hypothesis, while not definitively proven, has been invoked to explain the relatively high transfection efficiency of other proton-sponge-type materials, such as lipopolyamines [15, 16], PAMAM dendrimers [17], and various imidazole-containing polymers [18–20]. The original hypothesis proposed that polyethylenimine (PEI) buffering in lysosomes induced osmotic rupture and subsequent escape [21]. Although the proton sponge hypothesis based on the findings of a lack of lysosomal involvement is challenged in PEI-mediated gene transfer, a version of this hypothesis, whereby PEI buffering induces osmotic rupture in endosomes prior to fusion with lysosomes [15, 22], is consistent with the findings of Godbey et al. [23]. The pH of D-glucosamine hydrochloride is acidic (3.5-4.5). This acidic condition supports the proton sponge hypothesis involving escape from endosomes and lysosomes due to QD labeling for long periods. The new polycationic function of D-glucosamine (proton sponge hypothesis: escape from the degradative lysosomal trafficking pathway) is useful and meaningful for cell biology. This nanoimaging technology is therefore indispensable for investigating the distribution of bioactive materials, including applications in medical diagnosis.

## 5. Conclusion

This is the first study to investigate the uptake of carboxylated QDs conjugated with D-glucosamine into cultured osteoblastic cells. The interesting findings of this study include the escape of quantum dots from lysosomes in the cytoplasm, as confirmed with the merging of both QD fluorescence and specific fluorescent staining of lysosomes in the cytoplasm.
